# Parasympathetic Response Profiles Related to Social Functioning in Young Children with Autistic Disorder

**DOI:** 10.1155/2013/868396

**Published:** 2013-09-30

**Authors:** Stephen J. Sheinkopf, A. Rebecca Neal-Beevers, Todd P. Levine, Cynthia Miller-Loncar, Barry Lester

**Affiliations:** ^1^Department of Psychiatry and Human Behavior, Warren Alpert Medical School of Brown University, Providence, RI 02912, USA; ^2^Department of Pediatrics, Warren Alpert Medical School of Brown University, Providence, RI 02912, USA; ^3^Brown Center for the Study of Children at Risk, Women & Infants Hospital, 101 Dudley Street, Providence, RI 02905, USA; ^4^Department of Psychology, University of Texas at Austin, 108 E. Dean Keeton Street, Austin, TX 78712, USA

## Abstract

Psychophysiology studies of heart rate and heart rate variability can be employed to study regulatory processes in children with autism. The objective of this study was to test for differences in respiratory sinus arrhythmia (RSA; a measure of heart rate variability) and to examine the relationship between physiologic responses and measures of social behavior. Participants included 2- to 6-year-old children with Autistic Disorder and children without autism. Heart rate and RSA were derived from ECG recordings made during a baseline period and then a stranger approach paradigm. Social and adaptive behavior was assessed by parent report. Groups did not differ in mean heart rate or RSA at baseline or in response to social challenge. However, children with autism were more likely to show a physiologic response to intrusive portions of the stranger approach than to less intrusive portions of this procedure. Nonautistic children were equally likely to respond to intrusive and less intrusive social events. Within the autistic group, physiologic response to the intrusive stranger approach corresponded to higher ratings of social adaptive behaviors. These results suggest that physiologic responses to social challenge may help understand differences in social behavioral outcomes in children with autism.

## 1. Introduction

 Past research has suggested that children with autism show disruptions in autonomic responses to environmental stressors [1–5]. However, research has varied in terms of the ages of children included in studies, the nature of stimuli that constituted the observational protocols, and the resulting findings of physiologic responses in comparison to nonautistic individuals. Moreover, while some studies have examined relations between autonomic measures and measures of social and behavioral functioning (e.g., [5]), relatively little research has compared individual differences in physiologic and behavioral characteristics. Examinations of responses in relation to behavioral markers of symptom presentation, syndrome severity, and other aspects of behavioral functioning may help to reconcile some of the inconsistencies found in prior research and has potential implications for developing and refining assessments and treatments of autism. 

Cardiorespiratory measures, including heart rate, respiratory sinus arrhythmia (RSA), and more general measures of heart rate variability (HRV), have been widely used in studies of child psychopathology, including some research on autism. RSA, a phenomenon defined as periodic variation in heart rate due to respiratory activity, reflects central nervous system influences on heart rate. RSA is mediated by inhibitory signals to the sinoatrial node from brainstem nuclei via the vagus and reflects parasympathetic nervous system (PNS) influences on heart rate variability. Higher baseline RSA is thought to indicate a readiness to engage with or respond to environmental demands, including aspects of the social environment. On the other hand, changes in RSA in response to environmental challenge are thought to indicate active engagement with and attention to stimuli [6]. Decreases in RSA during social stressors may indicate adaptive attention to social information and thus may be related to positive social behavior and adaptation to social environmental demands. 

Two studies have investigated changes in HRV, during cognitive tasks. Althaus and colleagues [7] examined HRV during a cognitive challenge in children with autism. They found that children with autism and typically developing children did not differ on measures of baseline heart rate or baseline HRV. However, the children with autism failed to show task-associated decreases in HRV in comparison to nonautistic children. These response patterns did not differentiate autistic children with and without hyperactivity, but changes in HRV from baseline did correlate with measures of behavioral inflexibility in the children with autism. Toichi and Kamio [4] also reported that adolescents with autism failed to show decreases in HRV during a cognitive task. All individuals in the control sample showed task-related decreases in HRV. In contrast, the autistic group showed a variable response, with some children showing decreases in HRV and others showing an unexpected increase in HRV. The authors argued that this finding was indicative of hyperarousal and that some individuals with autism may have found the baseline condition more stressful than the condition where they were engaged in a task. An additional relevant interpretation of this observation is that focusing on the average levels of physiologic responses may mask important and potentially meaningful individual differences. 

Few studies have examined RSA or other measures of HRV in response to social events and stimuli. Althaus and colleagues [8] demonstrated qualitative differences between 8–12-year-old children with and without a diagnosis of Pervasive Developmental Disorder Not Otherwise Specified (PDD-NOS) on parasympathetic-related changes in HRV, but not on components of HRV modeled as sympathetic influences. Furthermore, the magnitude of these differences was greatest for a subgroup of children with PDD-NOS and comorbid hyperactivity and attention problems. In another study of higher functioning children with autism, Levine et al. [3] found no difference in RSA between autistic and nonautistic children during the Trier Social Stress Test [9]. There were also no differences at baseline and following the task. Bal et al. [10] found that 7–17-year-old children with autism without significant cognitive deficits had lower RSA when compared to controls at baseline. Within the autism group, those with higher RSA showed more rapid recognition of facial emotions than those with lower RSA. 

An additional and notable aspect of prior research is that there have been limited studies of autonomic measures of response and regulation in young children with autism. Corona et al. [11] reported that 3–5-year-old children with autism failed to show a heart rate response to the feigned distress of an examiner, as compared to a matched sample of children with developmental delays that showed an overall decrease in heart rate in response to the examiner's distress display. In related work, Sigman and colleagues [12] reported that both young children with autism and a matched group of children with developmental delays showed decreased heart rate when viewing a video of babies laughing or crying. While these studies did not examine measures of HRV such as RSA, they do suggest experimental design will affect the intensity, salience, or other aspects of the stimuli that can account for between-study discrepancies in findings. More recently, Patriquin et al. [13] reported a positive association between baseline RSA and both receptive language and children's sharing behaviors during play in a relatively high functioning sample of 4- to 7- year-old children with autism spectrum disorders.

 With these issues in mind, the goal of the present pilot study was to investigate group and individual differences in autonomic responses to graded social stimuli in young, relatively lower functioning children with autism and to determine whether individual differences in autonomic responses would be related to behavioral measures of competence. To this end, we recorded electrocardiograph (ECG) signals during a standardized stranger approach paradigm, during which a female examiner entered the room and progressively approached and interacted with the child. We hypothesized that children with autism would show a diminished or delayed RSA response to stranger approach compared to a matched comparison sample, and children who showed greater RSA responses to the social approach of the stranger (i.e., decreases in RSA) would have higher ratings of social behavioral competence. 

## 2. Materials and Methods

### 2.1. Participants

Participants included children with Autistic Disorder (AUT; *n* = 15) and children without autism (non-AUT; *n* = 8), group-matched on chronological age and nonverbal IQ. Autism diagnoses were determined by clinician best estimate diagnosis confirmed by above-threshold scores on the Autism Diagnostic Observation Schedule [14]. Participants ranged in age from 30 to 79 months and had variable levels of cognitive and language functioning. Cognitive developmental abilities were assessed by either the Differential Ability Scales (DAS) [15] or the Bayley Scales of Infant and Toddler Development, second edition [16], as was developmentally appropriate. In order to allow for some comparability of cognitive scores across children who received different measures, we group-matched participants on the basis of the DAS nonverbal IQ score or the Bayley Mental Development Index (MDI). Adaptive functioning, including social behavioral skills, was assessed using the Vineland Adaptive Behavior Scales [17]. The AUT group had lower Vineland Composite scores, as well as lower Daily Living and Socialization scores, than the non-AUT group. The variability in level of functioning in the children with autism is consistent with what is seen in the general population of children with autism spectrum disorders. Roughly half (47%) had cognitive standard scores (from the Bayley Scales or DAS) less than 75, and 80% had Communication standard scores on the Vineland less than 75 (i.e., more than 1.5 standard deviations below the normative sample means). The comparison group included some children with general developmental cognitive delays (63% with cognitive standard scores less than 75; 87% with Vineland Communication standard scores less than 75). There were no comparison children with developmental delays of known etiology (i.e., there were no children with Down Syndrome, fragile X, or other known genetic conditions).

Groups did not differ in other demographic characteristics. Descriptive data are presented in Table 1. Parents provided informed consent for study participation, and the research was approved by the hospital Institutional Review Board that provided oversight for the ethical treatment of human participants. 

### 2.2. Procedure

 We recorded ECG data from a 5-minute baseline period during which children were engaged in a quiet and nonchallenging tabletop activity (determined with consultation with the child's parent). This was followed by a stranger approach procedure similar to methods used in prior developmental research [18]. With the child seated at the table and his or her parent seated behind and slightly to the side, a female experimenter, unfamiliar to the child entered the room and then approached the child in two stages, each lasting 2 minutes. This resulted in an overall stranger approach procedure that was similar in length to the baseline. The comparison of a somewhat longer baseline to shorter stimulus conditions (i.e., 5 min. baseline versus 2 min. stages of the stranger approach) is consistent with prior psychophysiology research in this and other populations (e.g., [11]). 

During the procedure, children remained seated at a small table facing towards the door to the room. A developmentally appropriate set toys were on the table during the stranger approach procedure. At the first stage, that we will call the distal stranger approach, the examiner stood near the door to the room while talking with the child's parent (60 seconds) and moved to a mark about half way to the child and continued to chat with the parent (60 seconds; strangers were instructed to participate in “small talk” with the parent, and the parent was briefed beforehand so as to expect this). At the second stage, that we will call the proximal stranger approach, the examiner sat down diagonally across the table from the child and started to talk to the child in a friendly voice (60 seconds) and then leaned in toward the child, gently touching the child on the arms, while talking in a friendly but intrusive manner (60 seconds). The order of these events (baseline, distal stranger, proximal stranger) was the same for all participants. The overall intent of this behavioral assay was to present a friendly but increasingly intrusive social approach of a stranger. 

### 2.3. ECG Acquisition and Processing

 ECG data were acquired via three electrodes placed on the child's chest and abdomen. The ECG raw data were recorded, digitized, and stored on a computer hard drive using a system designed to coordinate the collection of physiologic data with video recordings in real time [19]. This system included a Hewlett Packard ECG monitor that recorded R-wave peaks to the nearest millisecond. The ECG monitor output included a set of second by second HR estimates and a separate set of R-R interval data that was used to calculate RSA. 

Further offline processing of the ECG signal involved data cleaning and calculation of RSA. Artifact detection and correction was performed using a series of automated algorithms. A moving confidence interval was used to detect RR intervals outside of expected values. Missed or spurious R-waves were in this way detected, flagged, and corrected by linear interpolation. Subsequent processing employed a time-series analysis and a moving polynomial filter to remove low frequency trends in the HR signal. This process removes periodicities in the ECG signal that are outside the frequency range of the respiratory cycle. The resulting measure is one of HRV in the frequency range of respiratory rate. The natural logarithm of heart period variance in this frequency range is a measure of RSA. The computations for RSA utilized the time-series analysis and calculations developed by Porges et al. [20].

### 2.4. Statistical Analysis 

Using these methods, we summarized the mean heart rate (HR) and RSA values for the baseline and stranger approach conditions. We also calculated percent change values for RSA from baseline to distal and proximal stranger approach stages. Moreover, based on our hypotheses, we grouped children into those showing a decrease in RSA from baseline to the distal and proximal stranger conditions and those who did not show a change in RSA or who showed an increase in RSA (i.e., a change score ≥ 0). Changes in RSA were calculated as a percentage change from baseline. *t*-tests of means were used to compare groups of children on dependent measures of physiology and adaptive behaviors. Tests of the likelihood of children to show physiologic responses to the study conditions were examined with Chi-square tests and McNemar tests of within-subject likelihood of responses. 

## 3. Results

### 3.1. HR and RSA Differences by Condition

The AUT and non-AUT groups did not differ in mean HR or mean RSA at baseline or during distal or proximal stranger approach conditions. In addition, groups did not differ significantly on the percent change of RSA from baseline to the distal or proximal stranger conditions. These results are reported in Table 2. Individual HR and RSA data points are presented in Table 3. There was one participant in AUT group with high levels of artifact in the RSA signal during stranger approach. The data from this procedure for this child was excluded. Artifactual HR data were found for 3 AUT group children and 2 non-AUT group children. These data points were also excluded from analyses. 

### 3.2. Differences in Likelihood to Show an RSA Response

While the groups did not differ in the mean levels of RSA or percent RSA change, there were individual differences in responses across participants. Within the AUT group, 3 of 14 children with RSA stranger approach data (21%) showed an RSA decrease in response to the distal stranger condition, whereas 9 of these 14 children (64%) showed an RSA decrease in response to the proximal stranger condition. Within the non AUT group, 3 of 8 children (38%) showed an RSA decrease in response to the distal stranger condition, whereas 4 of these 8 children (50%) showed an RSA decrease in response to the proximal stranger condition. Chi square tests of likelihood for responses by group were not significant for either the distal or proximal conditions (Fishers exact tests). However, when comparing likelihood of response across the conditions but within each group, the AUT group was found to be more likely to show an RSA decrease in response to the proximal stranger approach than to the distal stranger approach (McNemar test, *P* = .03). Specifically, 6 of the 11 (55%) AUT children who did not show a response to the distal stranger approach showed an RSA decrease to the proximal stranger approach, and all 3 AUT children who showed an RSA decrease to the distal stranger also showed a proximal stranger-related RSA decrease. This difference in likelihood of response between conditions was not found in the non AUT group.

### 3.3. Comparison of Responders versus Nonresponders

We classified the AUT subjects into RSA responders versus nonresponders on the basis of RSA decreases from baseline to the proximal stranger condition (i.e., a “responder” was a child whose RSA was lower during the stranger approach than that during baseline). These groups were compared on Vineland Scales standard scores. One subject had missing Vineland data. Therefore, there were 9 responders and 5 non-responders included in these comparisons. AUT children classified as responders had higher Vineland Socialization standard scores than non-responders, *t* (11) = 2.4, *P* = .03. Responders did not differ from non-responders on the other Vineland domain scores. These results are reported in Table 4. 

## 4. Discussion

This study revealed differences in patterns of RSA responses to social events of varying degrees of intensity in children with autism compared to controls. Specifically, children with autism were more likely to show a response (a decrease in RSA from baseline) during the intrusive “proximal” stranger approach than during the initial and less intrusive entry of the stranger into the room. This difference in likelihood of response was not seen in the nonautistic group. Because decreases in RSA reflect active response and engagement with the environment, this finding suggests that an increased level of intensity of stimulation or a degree of vigor of interaction is needed to elicit normative physiologic responses in children with autism. Importantly, and consistent with prior work [11], we did not see evidence for heart rate increases in the autistic versus nonautistic group in response to the intrusive stranger approach. Thus, where RSA responses are elicited, they do not appear to be indicative of a “fight-or-flight” sympathetic response to social stimuli. 

 Individual differences in responses to intrusive social events were associated with social functioning in the children with autism. Autistic children who were physiologically responsive to the proximal stranger approach had better social functioning as measured by the Vineland scales, a parent-report instrument. It is noteworthy that in the group of children with autism, responders and non-responders did not differ on other sections of the Vineland scales. This provides discriminant validity to our findings and suggests that patterns of autonomic responses specifically to people are indicative of levels of social attention that support social behavioral functioning in children with autism. Physiologic responses to social events may be related to behavioral functioning, but the nature of the observational context will affect whether meaningful individual differences are observed. This finding has implications for individualizing educational and behavioral strategies for subgroups of children with autism who are less reactive to the environment as indicated by attenuated RSA responses to social presses. For example, it may be beneficial to individually tailor the degree to which therapeutic interactions vigorously attempt to elicit social responses from children. A relevant question raised by these results is whether research on physiologic responses can be utilized to validate behavioral indicators, or whether physiologic monitoring can itself augment such individual treatment strategies. This can be seen as consistent with clinical approaches that include attention to managing arousal during treatment [21]. 

Young children with autism, however, did not differ in their level of RSA during a baseline condition and did not differ in RSA response during an increasingly intrusive stranger approach observation. This negative finding is perhaps related to the small sample size in this study. However, the small effect sizes of group differences and the variability of responses to stranger approach suggest that putative differences in RSA and similar measures of autonomic functioning may be relatively small for children with autism when compared to children matched on age and cognitive abilities. Interestingly, in terms of RSA differences, the largest effect size, although relatively small and statistically nonsignificant, was for the comparison of RSA during the initial distal stranger approach. Here, the children with autism showed a modest RSA increase from baseline, whereas the comparison group showed a modest decrease in RSA from baseline. This could suggest that novel social events, such as initial entry of a new person, are more salient for nonautistic children than for autistic children, although larger samples are needed to determine if such differences are statistically reliable. Some prior studies have found differences between older groups of children with autism and comparison subjects on measures of baseline and task-related heart rate variability. Thus, an additional explanation for our finding of no group difference is that the children observed in this study were fairly young. Because baseline RSA can be expected to increase with age in the early childhood years, group differences in levels of RSA may not be evident until this system matures and RSA approaches adult-like levels [22]. It is also noted that whereas some children showed decreases in RSA relative to baseline, others showed increases in RSA. The interpretation of such increases is unclear, but it is possible that the increases in RSA during tasks or experimental conditions could be attributable to individual differences in how children responded to initial baseline conditions as much as their parasympathetic response to challenge. 

These results are preliminary in nature, and there are several limitations to this study that should be discussed. The sample size in this study was small, limiting our statistical power to detect significant differences between groups. A related challenge to this work is the difficulties, both practical and conceptual in nature, in matching on key characteristics. Subject matching in autism research is a common but challenging practice [23]. One of the emerging complexities with subject matching is the heterogeneity of individuals with autism. In the present study, we matched the groups on age and IQ. However, the variability in these characteristics, while similar in the two groups, may add measurement error. Thus, future research may yield different results with more homogenous groupings on age and level of functioning. 

## 5. Conclusions

In this study we report on individual differences in RSA responses to social events in relation to parent-reported social adaptive functioning in children with autism. While children with autism did not differ from controls in RSA reactivity to a social approach paradigm, they were more likely to demonstrate decreased RSA in response to more proximal social interactions. This reactivity to proximal social interactions was related to better social functioning, indicating that individual differences in social competence, along with intensity of a social interaction can discriminate subgroups of children with autism who may show differential responses to graded social interactions. Findings such as these have implications for treatment and suggest that individual differences in thresholds for social responses may indicate therapeutic strategies matched to such characteristics. Clearly, replication and extension of these findings are needed, and future research should also focus on the stability of such response styles within individuals. More generally, and in conclusion, this pilot research suggests that a focus on individual differences in psychophysiologic responses may help to clarify inconsistencies in prior research and would have implications for understanding individual differences in treatment outcomes in this population. 

## Figures and Tables

**Table 1:** Characteristics of the children in the autistic (AUT) and nonautistic (non-AUT) groups.

	AUT	non-AUT	*P*
Age—mean (SD)	4.3 (1.2)	3.6 (1.0)	ns
Gender (*n*)			ns
Male	12	7	
Female	3	1	
Race/ethnicity (*n*)			ns
White	15	7	
Black	0	1	
SES-Hollingshead (*n*)			ns
Mid/high (levels I–III)	15	7	
Low (levels IV-V)	0	1	
Vineland scores—mean (SD)			
Communication	64.1 (14.6)	73.4 (16.9)	ns
Daily living	56.6 (5.2)	71.4 (10.9)	<.01
Socialization	57.4 (5.1)	74.4 (12.9)	<.01
Motor	66.2 (11.5)	76.4 (15.6)	ns
Vineland composite	56.3 (6.1)	69.3 (14.8)	<.01
IQ estimate—mean (SD)	71.9 (28.7)	75.0 (18.7)	ns

**Table 2:** Descriptive data for heart rate (HR) and respiratory sinus arrhythmia (RSA) measures for the autistic (AUT) and nonautistic (non-AUT) groups.

Measure	AUT	non-AUT	Effect size |*d*|	*P*
	Mean (SD)	Mean (SD)		
HR				
Baseline	108.93 (13.96)	109.88 (15.43)	0.06	ns
Distal stranger	108.32 (15.34)	109.92 (13.34)	0.11	ns
Proximal stranger	106.06 (16.87)	106.71 (12.87)	0.04	ns
RSA				
Baseline	4.87 (1.55)	4.87 (0.92)	<0.01	ns
Distal stranger	5.33 (1.35)	4.87 (1.07)	0.37	ns
Proximal stranger	4.96 (1.24)	4.76 (1.11)	0.17	ns

**Table 3:** Individual data values for RSA and HR for AUT and non-AUT participants.

Subject	RSA values			HR values		
	Baseline	Distal stranger	Proximal stranger	Baseline	Distal stranger	Proximal stranger
AUT group						
AUT1	4.83	5.05	4.58*	118.93	123.58	122.75
AUT2	8.19	8.51	8.19	90.91	86.02	91.01
AUT3	5.31	5.66	4.66*	97.35	99.35	102.49
AUT4	5.86	5.96	4.70*	100.62	97.33	86.74
AUT5	5.74	6.01	6.36	92.23	96.15	92.28
AUT6	6.46	5.99*	5.03*	88.88	90.88	77.99
AUT7	5.72	5.83	5.25*	112.27	110.73	111.47
AUT8	3.12	5.91	5.14	115.67	120.95	118.02
AUT9	3.64	4.26	4.81	124.57	117.79	112.85
AUT10	3.56	3.54	3.35*	125.99	122.55	124.45
AUT11	3.20	a	a	118.871	a	a
AUT12	4.64	4.38*	4.06*	120.92	125.87	124.44
AUT13	5.81	5.63*	5.65*	m	90.16	89.14
AUT14	2.13	2.89	3.22	m	126.87	125.13
AUT15	4.87	5.01	4.38*	m	m	m
non-AUT group						
Non-AUT1	4.65	4.37*	4.79	m	112.62	104.86
Non-AUT2	5.88	6.12	6.20	99.20	96.73	95.69
Non-AUT3	5.01	4.54*	4.30*	114.47	123.51	117.69
Non-AUT4	5.43	3.65*	3.96*	m	113.40	110.16
Non-AUT5	5.40	5.58	5.20*	99.77	103.61	96.41
Non-AUT6	5.59	6.52	6.26	91.80	86.34	87.59
Non-AUT7	3.59	4.30	4.43	131.40	121.38	125.30
Non-AUT8	3.41	3.85	2.97*	122.65	121.80	115.94

*indicates subjects categorized as having an RSA response defined as a decrease in RSA versus baseline.

a indicates missing values due to artifact in the ECG signal.

m indicates missing HR data during technical problem with the HR calculation for this subject.

**Table 4:** Adaptive behavior standard scores for AUT group participants classified as RSA responders versus non-responders to proximal stranger approach.

Vineland domain	Responders	Nonresponders	Effect size |*d*|	*P*
	Mean (SD)	Mean (SD)		
Communication	66.63 (15.81)	59.20 (14.36)	0.48	ns
Daily living skills	57.38 (5.78)	54.00 (2.45)	0.70	ns
Socialization skills	59.50 (4.90)	53.40 (3.51)	1.37	.03
Composite score	57.25 (5.95)	54.40 (7.30)	0.44	ns
